# Comparison of Spin-Flip TDDFT-Based Conical Intersection Approaches with XMS-CASPT2

**DOI:** 10.1021/acs.jctc.9b00917

**Published:** 2020-04-17

**Authors:** Max Winslow, Warren B. Cross, David Robinson

**Affiliations:** Department of Chemistry and Forensics, School of Science and Technology, Nottingham Trent University, Clifton Lane, Nottingham NG11 8NS, United Kingdom

## Abstract

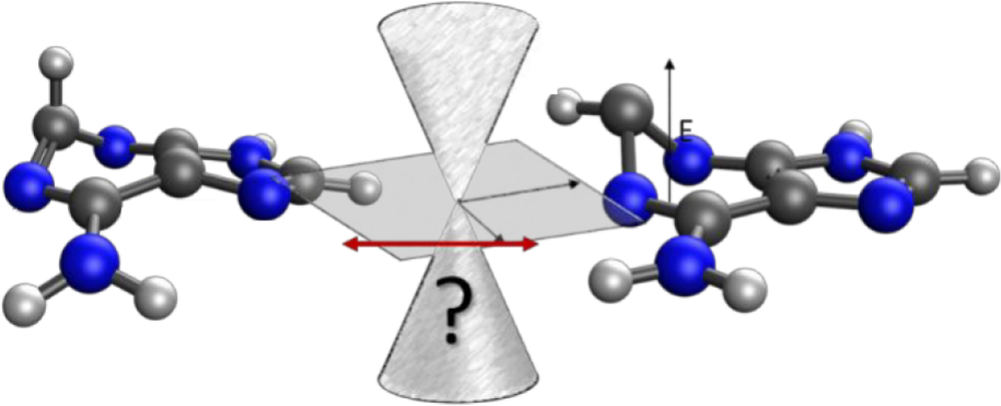

Determining conical intersection geometries is of key importance to understanding the photochemical reactivity of molecules. While many small- to medium-sized molecules can be treated accurately using multireference approaches, larger molecules require a less computationally demanding approach. In this work, minimum energy crossing point conical intersection geometries for a series of molecules have been studied using spin-flip TDDFT (SF-TDDFT), within the Tamm-Dancoff Approximation, both with and without explicit calculation of nonadiabatic coupling terms, and compared with both XMS-CASPT2 and CASSCF calculated geometries. The less computationally demanding algorithms, which do not require explicit calculation of the nonadiabatic coupling terms, generally fare well with the XMS-CASPT2 reference structures, while the relative energetics are only reasonably replicated with the MECP structure as calculated with the BHHLYP functional and full nonadiabatic coupling terms. We also demonstrate that, occasionally, CASSCF structures deviate quantitatively from the XMS-CASPT2 structures, showing the importance of including dynamical correlation.

## 1 Introduction

Photochemical processes are ubiquitous in nature and form the backbone of many important chemical processes. In nature, the photoinduced isomerization of retinal forms the basis of vision, while absorption of light by chlorophyll is important in photosynthesis. In terms of man-made processes, dye-sensitized solar cells,^1^ fluorescent molecular probes (a full literature review is beyond the scope of the current work; see, e.g., ref (2)), chemosensors,^3^ energy transfer cassettes,^4^ photodynamic therapy agents,^5^ and tunable laser dyes^6−8^ are just a handful of the many applications of photochemistry. Key to the correct computational description of absorption of radiation by such molecules is the transition dipole moment.^9^ Upon absorption, the molecule may relax to the ground electronic state via different routes: emission, phosphoresence, and radiationless decay (via a conical intersection). The last of these relaxation methods provides an extreme test of the robustness of computational methods, since at the conical intersection there are (at least) two degenerate electronic states, and the Born–Oppenheimer approximation breaks down (see, e.g., refs (10) and (11)).

Many studies have employed the CASSCF method to explore the excited state pathways (including conical intersections); there are far too many to include here. There are numerous examples also where TDDFT-based methods have been applied to such problems. In general, TDDFT results have been compared to CASSCF (where possible), with mixed accuracy. Minezawa and Gordon^12^ compared spin-flip (SF) TDDFT minimum energy crossing point (MECP) conical intersection geometries of ethene with those determined at the MRCI and MS-CASPT2 levels of theory, with SF-TDDFT correctly predicting the three conical intersection geometries determined by the multireference methods. Filatov^13^ studied the dependence of the choice of the density functional upon MECP geometry compared with various multireference approaches (CASSCF, CASPT2, MRCI), concluding that the BHHLYP hybrid functional performed the best, while popular contemporary functionals, such as M06-2X, perform relatively poorly. Nikiforov et al.^14^ studied a group of small organic molecules, using the restricted ensemble-referenced Kohn–Sham (REKS) approach, comparing their results with MR-CISD calculated geometries. They determined average RMSD differences from the MR-CISD geometries of ∼0.1 Å, although the underlying MCSCF wave functions for some of the molecules had reduced active spaces due to technical limitations. Zhang and Herbert^15^ compared SF-TDDFT calculated MECP conical intersection geometries of 9H-adenine to MR-CIS results,^16^ noting that the difference between the two methods was nearly indistinguishable. Recently, Segarra-Marti et al.^17^ studied the excited state decay of uracil and thymine cations, while including dynamical correlation using extended multistate CASPT2 (XMS-CASPT2).^18^ They found that inclusion of dynamical electron correlation resulted in the separation of the energy levels of a “3-state” conical intersection, giving a different geometry and energy.

While it is desirable to use the highest-level theory possible to determine MECP geometries, for systems of interest in both chemistry and biology, it is not always possible to use multireference approaches. In the current study, we compare MECP conical intersection geometries calculated using SF-TDDFT, both with and without explicit calculation of nonadiabatic coupling terms, with both CASSCF and XMS-CASPT2. Our primary motivation is to determine the accuracy of SF-TDDFT S_1_/S_0_ MECPs against XMS-CASPT2 for a set of medium-sized molecules, with particular emphasis on methods that do not require explicit computation of the nonadiabatic coupling terms. It is therefore useful to establish a protocol for the optimization of MECP geometries of larger molecules that treat electron correlation sufficiently. Boggio-Pasqua and Bearpark have recently investigated a similar approach to radical polycyclic aromatic hydrocarbons.^19^ The rest of the paper is organized as follows: in section 2, we present an overview of the background theory of the approaches used; in section 3, we give the computational details; in section 4, we present the results of our comparisons, and in section 5 we give our conclusions.

## 2 Background Theory

We here present a brief overview of the background theory to the methods involved, in order to fully understand the key differences between each approach.

### 2.1 Branching Planes

In the vicinity of a conical intersection, two (or more) electronic states become degenerate, and the Born–Oppenheimer approximation breaks down. Consider the case where two electronic states, *J* and *K*, become degenerate; given *N*_int_ internal degrees of freedom, the (*N*_int_–2)-dimensional space is known as the seam space, in which the two electronic states are degenerate, and the remaining two degrees of freedom are called the branching space. Within the branching space, the degeneracy of the Born–Oppenheimer surfaces is lifted by an infinitesimal shift in nuclear coordinates. The branching space is spanned by two vectors, **g**_*JK*_ and **h**_*JK*_. The first is evaluated simply as the difference in gradient vectors of the two Born–Oppenheimer electronic states:
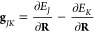
The second vector, **h**_*JK*_, is known as the nonadiabatic coupling vector and is defined as
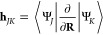
The derivative coupling vector, **d**_*JK*_, can then be calculated as
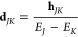
The topology of the seam space is determined by the relative orientation and magnitude of the two vectors **g**_*JK*_ and **h**_*JK*_.

### 2.2 Multireference Approaches

Assuming an appropriately chosen active space, the CASSCF and/or XMS-CASPT2^20−23^ approach can be used to calculate both **g**_*JK*_ and **h**_*JK*_ analytically, using state-averaged wave functions (over the Born–Oppenheimer electronic states of interest). Further details of the multireference calculations are given below.

### 2.3 DFT/TDDFT Approaches

#### 2.3.1 Brillouin’s Theorem

If one considers the explicit form of the Hamiltonian for a calculation of a conical intersection
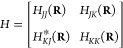
then, for a CIS calculation, the off-diagonal term H_*JK*_(**R**) must be zero when an S_1_/S_0_ conical intersection is calculated, due to Brillouin’s theorem (this is not strictly true for TDDFT but is, in general, observed for functionals typically used with exact Hartree–Fock exchange). As a result, the coupling matrix elements of **h**_*JK*_ vanish, and the degeneracy is only over one degree of freedom, resulting in an incorrect topology of the conical intersection. No such condition arises for two excited states becoming degenerate. One approach used to tackle this problem is SF-TDDFT,^24^ in which the reference state (equivalent to the “ground-state”) has a different spin multiplicity to the target states, hence the “ground-state” is also treated as an excited state, thus both **g**_*JK*_ and **h**_*JK*_ determine the topology around the conical intersection.^25^ SF-TDDFT approaches can be used to calculate analytic derivative couplings.^25^

#### 2.3.2 Penalty Function Optimization

Where analytic derivative couplings cannot be calculated, or where their calculation is expensive, more approximate methods can be used. The first considered here is the penalty-constrained optimization algorithm of Martínez et al.^26^ In this approach, minimization of the objective function
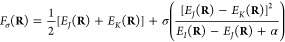
is performed, where α is a parameter employed to avoid singularities, and σ is a Langrange multiplier. The minimization of the penalty function is performed in an iterative manner for increasing values of σ.

#### 2.3.3 Branching Plane Update

The second approximate approach considered in this study is the branching-plane update algorithm of Morokuma et al.^27^ The mean energy gradient, **G**_mean_, is defined as
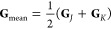
and the normalized difference gradient is given as
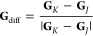
A projection vector, **P**, can then be defined as
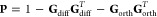
where **G**_orth_ is a vector orthogonal to **G**_diff_ and is an approximation to the derivative coupling vector. Finally, the gradient of the objective function is defined as
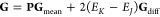


## 3 Computational Details

XMS-CASPT2 calculations were performed for the molecules given in Table 1 (including active spaces). These molecules were chosen as representative of molecular structures found in molecular probes of biological function, including a model of long unsaturated lipid tails (2,4,6-octatriene). The basis sets were chosen to match those used in the references provided (Table 1). The S_0_, S_1_, and S_1_/S_0_ MECP geometries were optimized, using an average over the first two singlet excited states (in *C*_1_ symmetry). The S_1_ excited state geometry was used as an initial guess for the conical intersection geometry. In the absence of convergence, a small “kink” was added to the molecular structure to aid convergence by avoiding any limiting symmetry constraint (in the case of a ring structure, the ring was puckered by moving one atom 0.1 Å out of the plane of the ring). In all cases, the MECP geometries to be found were those that match the literature references given in Table 1. A real shift of 0.2 au was used in all XMS-CASPT2 computations. Density fitting was used in all XMS-CASPT2 calculations, employing the TZVPP-JKFIT density fitting basis set, except for fulvene, where the cc-pVDZ-JKFIT set was employed. MECP geometries were determined using the same gradient projection algorithm as for SF-TDDFT.^28^ All XMS-CASPT2 geometry optimizations were performed using the BAGEL software.^29,30^ CASSCF conical intersection geometry calculations were performed with the Molpro 2015.1 software^28,31^ without the use of density fitting. In all cases, we were comparing SF-TDDFT approaches to XMS-CASPT2 for well-known MECPs, not trying to identify novel MECPs.

**Table 1:** Molecules Considered in This Work

molecule	active space	basis set(s)
fulvene^32^	(6,6)	cc-pVDZ
4ABN^33^	(10,9)	6-31+G(d)
5FC^34^	(8,7)	6-31G(d)
9H-adenine^35^	(12,10)	6-31G(d,p)
2,4,6-octatriene^36^	(6,6)	6-31+G(d)
azomethane^37^	(6,4)	6-31G(d)
azoxymethane^37^	(6,4)	6-31G(d)
phenol^38^	(8,7)	6-31G(d,p)
SMAC^39^	(8,8)	6-31G(d,p)

SF-TDDFT calculations were performed using the BHHLYP^40,41^ and ωB97X^42^ functionals and the basis sets given in Table 1. The BHHLYP functional has 50% Hartree–Fock exchange; such functionals are noted as successful within the spin-flip methodology.^24,43^ The ωB97X functional was chosen as an example of a contemporary range-separated hybrid functional that performs well for a variety of applications.^44^ MECP geometries were determined by analytical calculation of the nonadiabatic coupling matrix elements as discussed in section 2.3.1,^25^ using the gradient projection algorithm of Bearpark et al.^28^ We denote this as NAC for brevity. The penalty-constrained optimization approach (discussed in section 2.3.2; here defined as PC)^26^ and branching-plane (discussed in section 2.3.3; here denoted as BP)^27^ algorithms were used to determine MECP conical intersection geometries using SF-TDDFT without the explicit calculation of the nonadiabatic coupling terms. The spin-flip approach was used to determine the “reference” TDDFT state, as discussed in section 2. Each of the approaches considered here was performed within the Tamm-Dancoff Approximation,^45^ due to restrictions in the implementation of the SF-TDDFT methodology. For the 5FC and azomethane molecules, convergence to the MECP structures using each of the SF-TDDFT approaches was poor. In these cases, increasing the basis set to 6-31G(d,p) and optimizing to the MECP geometry using NAC-BHHLYP gave a good starting geometry for each of the SF-TDDFT approaches with the 6-31G(d) basis set. The DFT and SF-TDDFT calculations were performed with the Q-Chem 5.0 software suite.^46^

## 4 Results and Discussion

In this section, we briefly compare each molecule individually with the calculated XMS-CASPT2 MECP geometries, before giving a more general discussion of the performance of the SF-TDDFT-based methods.

### 4.1 Fulvene

Fulvene has been widely studied as a benchmark for calculations of the MECP conical intersection between the S_0_ and S_1_ electronic states.^32^ Given in Table 2 are selected geometrical parameters for the stable MECP.^32^ Qualitatively, each structure calculated using CASSCF and SF-TDDFT methods matches the XMS-CASPT2 reference structure well. The calculated bond lengths of the CASSCF structure are within 0.01 Å of the XMS-CASPT2 structure, while the largest deviation for the SF-TDDFT methods is 0.04 Å. The relative energetics are given in Table S2 (Supporting Information). Each of the methods using BHHLYP is very similar, with a deviation from the XMS-CASPT2 energies of ∼0.5 eV for the difference between the S_0_ minimum and MECP energies.

**Table 2:** Selected Geometrical Parameters for the S_1_/S_0_ MECP of Fulvene with the cc-pVDZ Basis Set^b^

	BHHLYP			ωB97X				
parameter	NAC	PC	BP	NAC	PC	BP	CASSCF^a^	XMS-CASPT2
C1–C2	1.396	1.395	1.395	1.388	1.389	1.415	1.410	1.417
C1–C4	1.473	1.473	1.475	1.485	1.483	1.480	1.461	1.471
C3–C5	1.474	1.473	1.473	1.484	1.483	1.484	1.460	1.471
C4–C5	1.340	1.340	1.339	1.344	1.345	1.358	1.371	1.377
C2–C6	1.464	1.464	1.464	1.476	1.475	1.458	1.481	1.477
H7–C–C–C3	–76.9	–75.5	–76.1	–68.0	–67.8	–76.8	–58.7	–67.5
H7–C–C–H8	179.1	180.0	179.2	180.0	179.8	173.4	171.3	180.0

a Taken from ref (32). (NAC – full nonadiabatic coupling terms calculate; PC – penalty constrained algorithm; BP – branching plane update method. See section 2 of the main text for full details.)

b Atom numbering taken from Figure 1.

### 4.2 4ABN

Groups studying the photochemistry of 4ABN (and related amino-substituted benzonitriles) are primarily interested in the S_2_/S_1_ conical intersection, as this plays a pivotal role in the presence or absence of dual fluorescence bands in polar/nonpolar solvents.^33^ Shown in Figure 2 is the XMS-CASPT2 S_1_/S_0_ MECP geometry for 4ABN, with selected geometrical parameters given in Table 3. The XMS-CASPT2 and CASSCF geometries both exhibit a “boat-like” conformation, with the C≡N and −NH_2_ groups both pointing away from the plane of the ring, while each of the SF-TDDFT methods has the −NH_2_ group nearly planar with the ring. In particular, the −NH_2_ group is completely planar for BP-ωB97X. The relative energetics are given in Table S3. Each of the SF-TDDFT approaches, except PC-BHHLYP, has a deviation from the XMS-CASPT2 relative energies of ∼0.8 eV (for the MECP).

**Figure 1:**
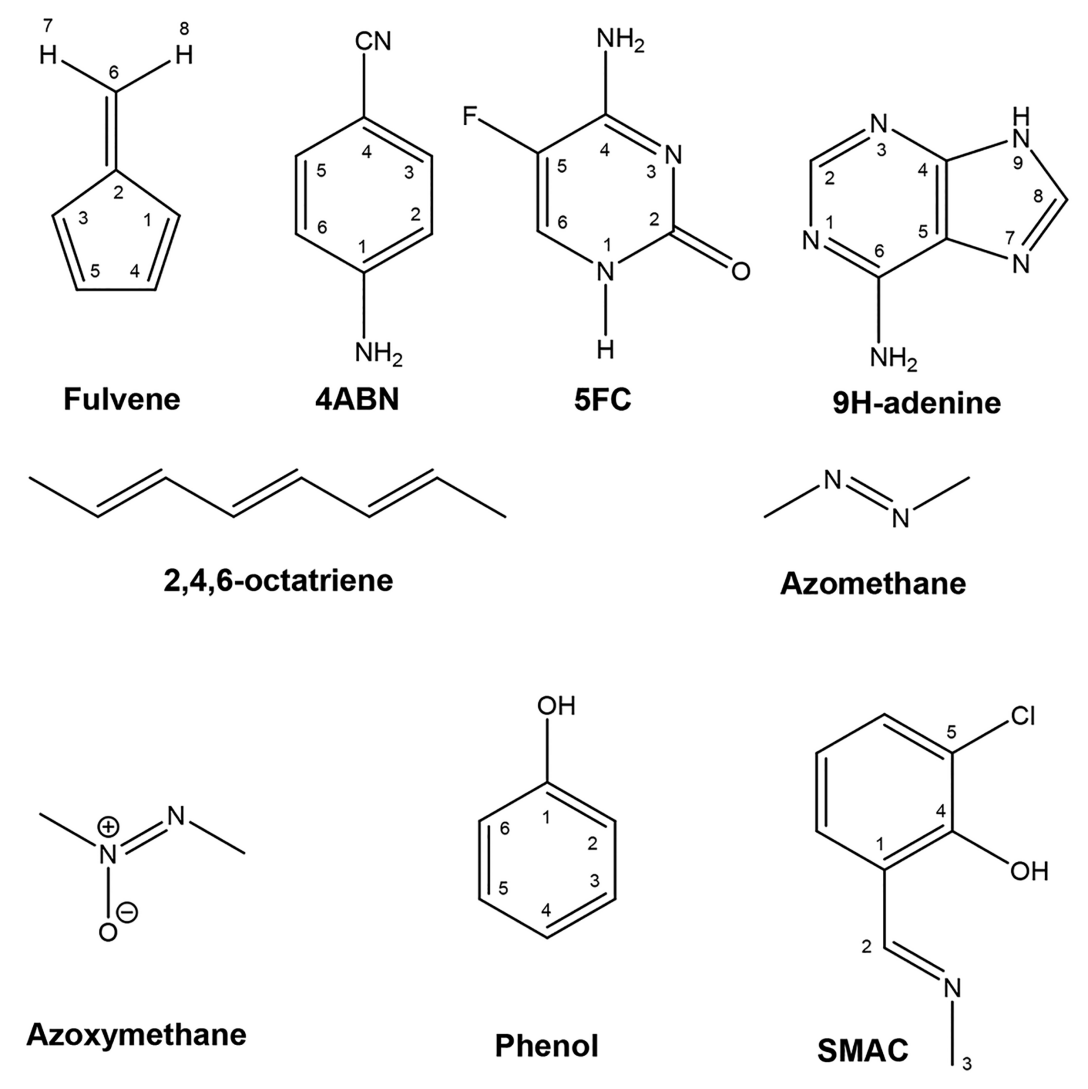
Schematic representation and numbering system used for the molecules considered in this work.

**Figure 2:**
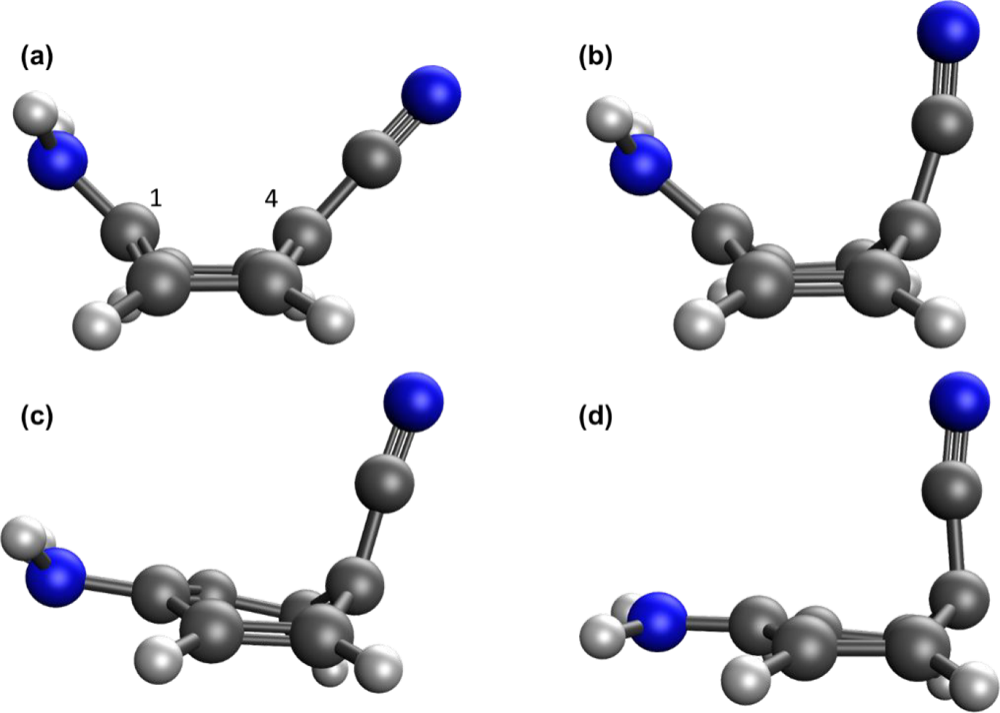
Calculated geometries for the S_1_/S_0_ MECP of 4ABN: (a) XMS-CASPT2, (b) CASSCF, (c) NAC-ωB97X, and (d) BP-ωB97X. Carbon atoms are gray, and nitrogen atoms are blue. Selected atom numbers are given in (a).

**Table 3:** Selected Geometrical Parameters for the S_1_/S_0_ MECP of 4ABN with the 6-31+G(d) Basis Set^a^

	BHHLYP			ωB97X				
parameter	NAC	PC	BP	NAC	PC	BP	CASSCF	XMS-CASPT2
C1–N (amine)	1.394	1.400	1.367	1.379	1.400	1.333	1.345	1.362
C4–C	1.421	1.422	1.421	1.406	1.422	1.436	1.421	1.398
C–N (cyano)	1.143	1.143	1.142	1.161	1.143	1.165	1.152	1.191
C2–C3	1.430	1.439	1.442	1.462	1.439	1.366	1.347	1.357
C5–C6	1.348	1.348	1.344	1.340	1.348	1.345	1.347	1.346
C2–C3–C4	108.4	108.5	108.6	107.2	108.5	114.7	112.5	110.2
C3–C4–C5	116.0	115.9	115.7	114.1	115.9	110.3	118.9	117.8
C4–C5–C6	110.1	110.1	110.5	112.5	110.1	114.5	112.5	110.1
H–C3–C4–C	80.5	68.5	75.0	61.9	68.5	104.6	34.3	59.2
H–N–C1–C2 (cis)	–21.3	–22.0	–22.1	–17.8	–22.0	–11.6	–20.7	–21.9

a Atom numbering taken from Figure 1.

### 4.3 5FC

**Figure 3:**
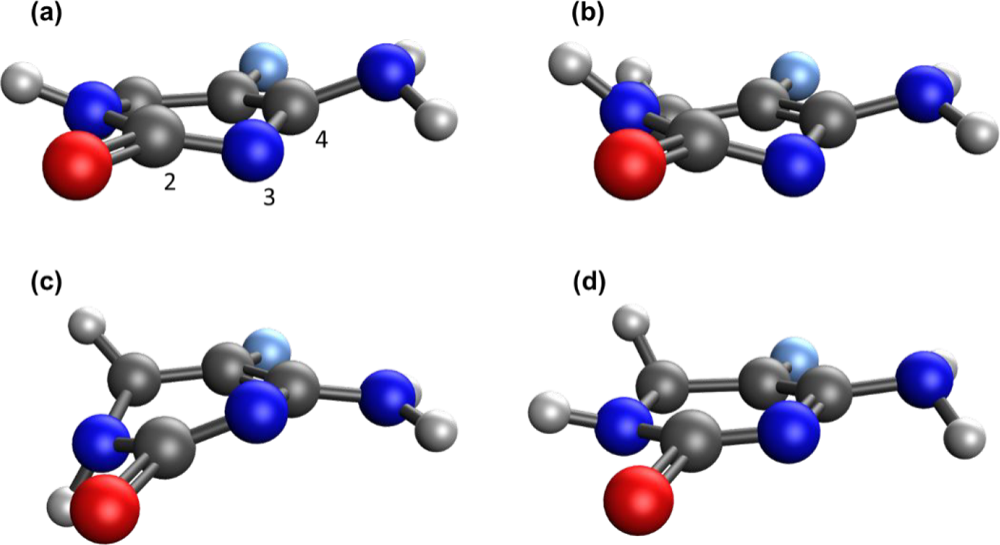
Calculated geometries for the S_1_/S_0_ MECP of 5FC: (a) XMS-CASPT2, (b) BP-ωB97X, (c) PC-ωB97X, and (d) NAC-ωB97X. Carbon atoms are gray, nitrogen atoms are blue, oxygen atoms are red, and fluorine atoms are light blue. Selected atom numbers are given in (a).

Blancafort et al. studied the photophysics of both cytosine and 5-fluorocytosine, characterizing both the S_1_/S_0_ and S_2_/S_1_ conical intersections to determine the nonradiative decay pathway.^34^ Shown in Figure 3 is the XMS-CASPT2 S_1_/S_0_ MECP geometry, along with geometrical parameters (Table 4). The CASSCF geometry shows reasonable agreement with the XMS-CASPT2 geometry, although the C–H and N–H bonds remain planar with the ring, unlike the XMS-CASPT2 geometry. The SF-TDDFT methods have qualitative deficiencies in comparison to XMS-CASPT2; the nonplanar nature of the ring and functional groups is quite different than that observed for XMS-CASPT2 (see Figure 3). In particular, the C–O bond is too long for each of the SF-TDDFT approaches. Qualitatively, the NAC approach most closely resembles the XMS-CASPT2 geometry; both the penalty constrained and branching plane update algorithms deviate further from the XMS-CASPT2 geometry. The relative energetics are given in Table S4. Where the MECP geometries are qualitatively correct, the relative energetics are within 0.4 eV of the XMS-CASPT2 energies.

**Table 4:** Selected Geometrical Parameters for the S_1_/S_0_ MECP of 5FC with the 6-31G(d) Basis Set^a^

	BHHLYP			ωB97X				
parameter	NAC	PC	BP	NAC	PC	BP	CASSCF	XMS-CASPT2
C4–N	1.383	1.383	1.383	1.396	1.357	1.372	1.389	1.389
C2–O	1.471	1.471	1.471	1.484	1.471	1.494	1.414	1.411
N1–H	1.001	1.001	1.001	1.009	1.018	1.003	0.997	1.016
C5–F	1.353	1.353	1.353	1.368	1.379	1.348	1.343	1.373
H–N–H	112.0	112.0	112.0	110.8	118.4	114.6	111.8	111.6
H–N–C4–C5	–17.1	–17.1	–17.1	–7.6	–8.7	–18.4	–21.2	–18.2
H–N1–C6–H	45.6	45.6	45.6	53.2	97.8	3.8	55.0	48.7

a Atom numbering taken from Figure 1.

### 4.4 9H-Adenine

**Figure 4:**
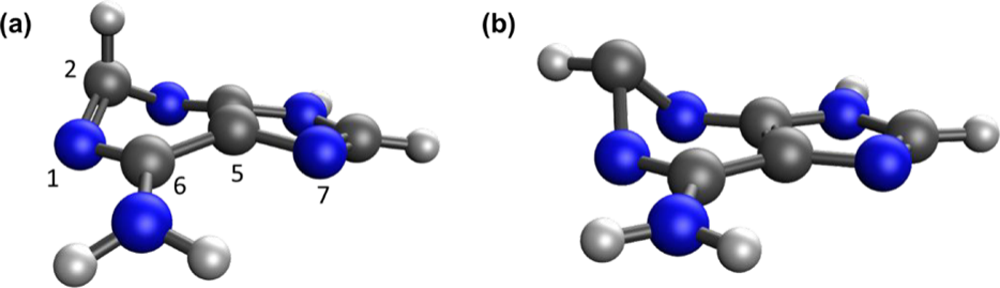
Calculated geometries for the MECP between the nπ* state and the ground state of 9H-adenine: (a) XMS-CASPT2 and (b) BP-BHHLYP. Carbon atoms are gray, and nitrogen atoms are blue. Selected atom numbers are given in (a).

Perun et al. discovered conical intersections for the radiationless decay mechanisms of the lowest energy ^1^nπ* and ^1^ππ* (^1^L_b_) electronically excited states of 9H-adenine, using CASSCF.^35^ Single-point CASPT2 energies calculated at the S_0_ equilibrium geometry predicted the ^1^ππ* (^1^L_b_) as the lowest singlet excited state and the ^1^nπ* as the S_3_ state, separated by the ^1^ππ* (^1^L_a_) state, in contrast to TDDFT and CIPSI results.^47^ Given in Tables 5 and 6 are the calculated conical intersection geometrical parameters, for the conical intersections between the ^1^ππ* (^1^L_b_) state and the ground state and the ^1^nπ* state and the ground state, respectively. The corresponding XMS-CASPT2 conical intersection geometries are shown in Figures S3(a) and S3(b), respectively. The SF-TDDFT approaches are qualitatively similar to the XMS-CASPT2 geometry, with the exception of the orientation of the C–H bond vector shown in Figure 4. There are some more significant quantitative differences (Tables 4 and 5); in particular, both CASSCF (for the ^1^ππ* state) and PC-ωB97X (for the ^1^nπ* state) exhibit large differences in the bond lengths compared to XMS-CASPT2.

**Table 5:** Selected Geometrical Parameters for the Conical Intersection between the ^1^ππ* State and Ground State of 9H-Adenine with the 6-31G(d,p) Basis Set^b^

	BHHLYP			ωB97X				
parameter	NAC	PC	BP	NAC	PC	BP	CASSCF^a^	XMS-CASPT2
N3–C2	1.277	1.368	1.390	1.396	1.363	1.393	1.285	1.396
C2–N1	1.404	1.504	1.288	1.295	1.322	1.298	1.402	1.319
C6–N1–C2–N3	68.1	53.6	64.9	66.0	67.2	65.3	66.0	31.1
C6–N1–C2–H	–139.9	–157.0	–165.4	–166.2	–165.8	–167.0	–142.3	–164.7

a Taken from ref (35).

b Atom numbering taken from Figure 1.

**Table 6:** Selected Geometrical Parameters for the MECP between the ^1^nπ* State and Ground State of 9H-Adenine Using the 6-31G(d,p) Basis Set^b^

	BHHLYP			ωB97X				
parameter	NAC	PC	BP	NAC	PC	BP	CASSCF^a^	XMS-CASPT2
N3–C2	1.446	1.470	1.470	1.489	1.517	1.449	1.407	1.435
C2–N1	1.426	1.444	1.444	1.417	1.400	1.439	1.390	1.413
C6–N1–C2–N3	74.3	83.9	83.8	74.4	70.8	63.7	67.6	64.4
C6–N1–C2–H	–171.2	–171.2	–171.2	–176.2	179.5	176.1	–84.1	–77.4

a Taken from ref (35).

b Atom numbering taken from Figure 1.

### 4.5 2,4,6-Octatriene

**Figure 5:**
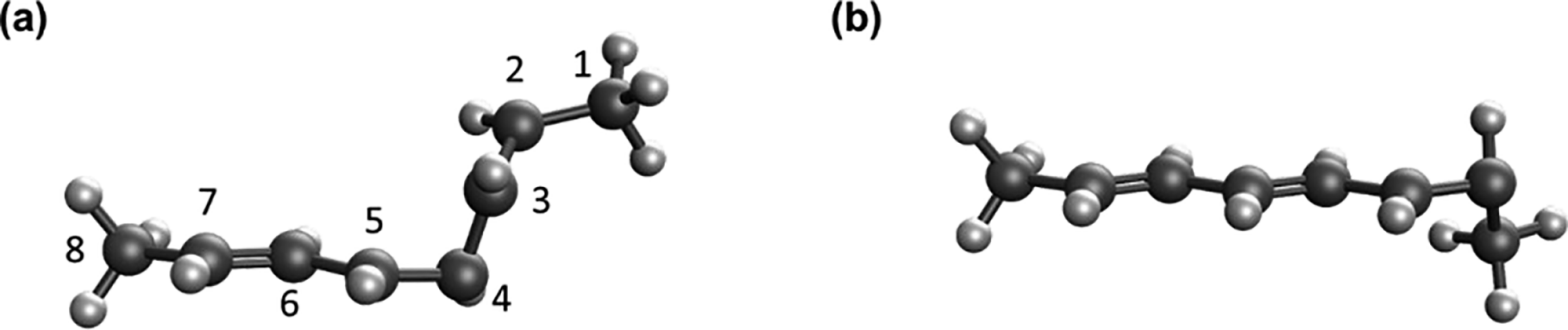
Calculated geometries for the S_1_/S_0_ conical intersection of 2,4,6-octatriene: (a) XMS-CASPT2 and (b) PC-ωB97X.

Chattopadhyay et al. characterized the S_1_/S_0_ conical intersection on the photoisomerization pathway of 2,4,6-octatriene at the CASSCF/6-31G(d) level, along with S_0_ and S_1_ equilibrium geometries.^36^ Shown in Figure 5 is the XMS-CASPT2 geometry, along with selected geometrical parameters in Table 7. Most of the methods qualitatively match the XMS-CASPT2 geometry, with reasonable quantitative accuracy. The exception is PC-ωB97X, which has a qualitatively different geometry (Figure 5). Despite numerous efforts, including starting from the XMS-CASPT2 geometry and selecting different electronic states in the spin-flip procedure for the MECP optimization, the geometry shown in Figure 5 was consistently obtained for this method. This suggests that the penalty constrained algorithm with the ωB97X functional is a poor approach to find such MECPs; indeed, when the full nonadiabatic coupling terms are included, with either ωB97X or BHHLYP, then good qualitative and quantitative agreement with XMS-CASPT2 is observed.

**Table 7:** Selected Geometrical Parameters for the S_1_/S_0_ MECP of 2,4,6-Octatriene, with the 6-31+G(d) Basis Set^a^

	BHHLYP			ωB97X				
parameter	NAC	PC	BP	NAC	PC	BP	CASSCF	XMS-CASPT2
C1–C2	1.494	1.494	1.494	1.494	1.560	1.494	1.504	1.499
C2–C3	1.444	1.441	1.444	1.441	1.419	1.441	1.464	1.453
C3–C4	1.404	1.399	1.406	1.399	1.445	1.399	1.427	1.408
C4–C5	1.445	1.448	1.446	1.448	1.351	1.448	1.466	1.464
C5–C6	1.407	1.417	1.407	1.417	1.452	1.417	1.427	1.419
C6–C7	1.359	1.364	1.357	1.364	1.340	1.364	1.365	1.378
C7–C8	1.489	1.496	1.485	1.496	1.494	1.496	1.501	1.497
C1–C2–C3	119.4	119.0	119.0	119.0	108.5	119.0	119.5	119.0
C1–C2–C3–C4	–100.8	–98.2	–102.0	–98.2	–57.0	–98.2	–103.9	–107.7
C2–C3–C4–C5	–127.4	–130.2	–126.9	–130.2	–177.3	–130.2	–118.2	–125.3
C3–C4–C5–C6	111.8	107.7	112.4	107.7	177.7	107.7	102.3	102.7

a Atom numbering taken from Figure 1.

### 4.6 Azomethane and Azoxymethane

Ghosh et al. studied the photoisomerization pathways of azomethane and azoxymethane, determining the S_1_/S_0_ conical intersection geometries for each, respectively.^37^ Shown in Figure S5 is the XMS-CASPT2 S_1_/S_0_ conical intersection geometry for azomethane, along with selected geometrical parameters in Table 8. Each of the SF-TDDFT-based methods qualitatively matches the XMS-CASPT2 geometry, with good quantitative agreement, including the relative energetics (Table S7).

**Table 8:** Selected Geometrical Parameters for the S_1_/S_0_ MECP of Azomethane, Using the 6-31G(d) Basis Set^b^

	BHHLYP			ωB97X				
parameter	NAC	PC	BP	NAC	PC	BP	CASSCF^a^	XMS-CASPT2
C1–N1	1.458	1.458	1.458	1.458	1.458	1.458	1.433	1.438
N1–N2	1.254	1.254	1.254	1.254	1.254	1.254	1.287	1.271
N2–C2	1.458	1.458	1.458	1.458	1.458	1.458	1.462	1.488
C1–N1–N2	120.2	120.2	120.2	120.2	120.2	120.2	130.9	114.4
N1–N2–C2	120.4	120.4	120.4	120.4	120.4	120.4	114.8	136.5
C1–N1–N2–C2	87.7	87.7	87.7	87.7	87.7	87.7	94.2	93.5

a Taken from ref (37).

b Atom numbering taken from Figure 1.

Given in Table 9 are selected geometrical parameters for azoxymethane (the XMS-CASPT2 S_1_/S_0_ conical intersection is shown in Figure S6). As for azomethane, the SF-TDDFT-based methods show generally qualitatively correct conical intersection geometries, apart from the N1–O1 bond. The C–N–N–C backbone exhibits more of a kink in comparison to the XMS-CASPT2 geometry, which has a dihedral angle close to 172°, while the poorest performing SF-TDDFT methods show an angle of ∼155° (BP-BHHLYP and BP-ωB97X).

**Table 9:** Selected Geometrical Parameters for the S_1_/S_0_ MECP of Azoxymethane, Using the 6-31G(d) Basis Set^b^

	BHHLYP			ωB97X				
parameter	NAC	PC	BP	NAC	PC	BP	CASSCF^a^	XMS-CASPT2
C1–N1	1.453	1.459	1.459	1.459	1.465	1.459	1.452	1.466
N1–N2	1.378	1.404	1.404	1.404	1.447	1.404	1.335	1.367
N1–O1	1.401	1.328	1.328	1.328	1.483	1.328	1.419	1.395
N2–C2	1.389	1.430	1.430	1.430	1.436	1.430	1.456	1.460
C1–N1–N2	109.1	112.0	112.0	112.0	106.1	112.0	114.2	112.3
N1–N2–C2	109.2	109.6	109.6	109.6	106.1	109.6	114.4	111.1
C1–N1–O1	107.4	112.6	112.6	112.6	95.4	112.6	112.6	110.3
C1–N1–N2–C2	172.5	155.1	155.1	155.1	163.3	155.1	178.6	172.1
C1–N1–O1–N2	116.3	117.4	117.4	117.4	108.9	117.4	118.7	115.6

a Taken from ref (37).

b Atom numbering taken from Figure 1.

### 4.7 Phenol

The photodissociation of the O–H bond of phenol has previously been studied, identifying the S_2_/S_0_ conical intersection as a critical point on the pathway (as well as an S_2_/S_1_ conical intersection).^38^ Given in Table 10 are selected geometrical parameters, while the XMS-CASPT2 geometry is shown in Figure S7. Most methods show good quantitative agreement with the XMS-CASPT2 geometry, except for BP-BHHLYP, which, while appearing qualitatively correct, exhibits significant quantitative differences from the XMS-CASPT2 geometry.

**Table 10:** Selected Geometrical Parameters for the S_1_/S_0_ MECP of Phenol, Using the 6-31G(d,p) Basis Set^a^

	BHHLYP			ωB97X				
parameter	NAC	PC	BP	NAC	PC	BP	CASSCF	XMS-CASPT2
C1–O	1.327	1.350	1.400	1.335	1.350	1.350	1.350	1.363
O–H	0.958	0.943	0.952	0.966	0.943	0.943	0.943	0.967
C1–C2	1.445	1.455	1.619	1.441	1.455	1.455	1.455	1.450
C2–C3	1.421	1.456	1.264	1.446	1.456	1.456	1.456	1.453
C3–C4	1.492	1.455	1.518	1.464	1.455	1.455	1.455	1.453
C4–C5	1.435	1.461	1.679	1.433	1.461	1.461	1.461	1.453
C2–C3–C4	84.2	84.5	101.8	81.9	84.5	84.5	84.5	83.0
H–O–C1–C2	162.9	165.9	152.2	174.9	165.9	165.9	165.9	168.5
O–C1–C2–H	–18.5	–31.1	–43.0	–27.4	–31.1	–31.1	–31.1	–28.5
C1–C2–C3–H	–176.4	–169.6	–176.2	–169.9	–169.6	–169.6	–169.6	–170.4

a Atom numbering taken from Figure 1.

### 4.8 SMAC

**Figure 6:**
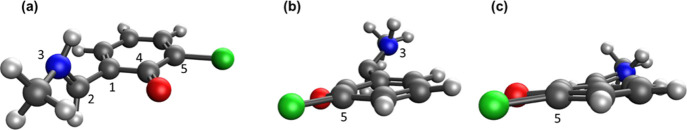
Calculated geometries for the ESIPT conical intersection of SMAC: (a) XMS-CASPT2, (b) BP-BHHLYP, and (c) BP-ωB97X. Carbon atoms are gray, nitrogen atoms are blue, oxygen atoms are red, and chlorine atoms are green. Selected atom numbers are shown for clarity.

Zhao et al. investigated the photoinduced isomerization mechanism of SMAC.^39^ In particular, they identified five MECP conical intersections using CASSCF. One of these is related to the excited-state intramolecular proton transfer (ESIPT) process, while the other four involve rotation (denoted as TW, for twist) around the C=N bond (see Figure 1). We retain the authors’ original naming convention for each of the conical intersection geometries here for convenience. For the ESIPT conical intersection, the XMS-CASPT2 geometry has the C–N–C(methyl) plane perpendicular to the plane of the aromatic ring (Figure 6). The PC-BHHLYP, PC-ωB97X, and NAC-BHHLYP methods all qualitatively match the XMS-CASPT2 geometry, albeit with some significant differences quantitatively, especially the O–H distance (Table 11). The best quantitative correlation occurs for the PC-ωB97X functional. The BP-BHHLYP geometry shows an angle of ∼45° between the two planes formed by the ring and the substituent, while the BP-ωB97X and NAC-ωB97X geometries exhibit near-planarity (Figure 6).

**Table 11:** Selected Geometrical Parameters for the “ESIPT” Conical Intersection of SMAC, Using the 6-31G(d,p) Basis Set^b^

	BHHLYP			ωB97X				
parameter	NAC	PC	BP	NAC	PC	BP	CASSCF^a^	XMS-CASPT2
C1–C2	1.471	1.446	1.540	1.419	1.489	1.419	1.460	1.468
C2–N	1.384	1.301	1.390	1.360	1.395	1.358	1.366	1.381
C5–Cl	1.723	1.722	1.727	1.777	1.711	1.776	1.733	1.722
O–H	3.624	3.318	4.191	4.928	3.614	4.879	3.307	3.193
N–H	1.003	1.012	1.006	1.013	1.014	1.018	0.995	1.012
C4–C1–C2–N	107.2	102.1	147.3	174.5	109.2	163.4	85.3	81.4
C1–C2–N–C3	–170.0	–167.9	–162.8	–177.3	–169.3	–175.9	–154.1	–145.0
H–O–C4–C1	–36.5	–36.5	–33.0	–11.1	–33.8	–19.1	–37.7	–38.8

a Taken from ref (39).

b Atom numbering taken from Figure 1.

The two XMS-CASPT2 conical intersection geometries, labeled “TWin1” and “TWin2”, show the N–CH_3_ perpendicular to the plane of the aromatic ring, with the methyl group pointing down or up, respectively (see Figures S8(b) and S8(c)). In all cases, the SF-TDDFT methods are qualitatively similar to the XMS-CASPT2 geometries, with fairly good quantitative agreement (Tables 12 and 13) for most geometrical parameters.

**Table 12:** Selected Geometrical Parameters for the “TWin1” Conical Intersection of SMAC, Using the 6-31G(d,p) Basis Set^b^

	BHHLYP			ωB97X				
parameter	NAC	PC	BP	NAC	PC	BP	CASSCF^a^	XMS-CASPT2
C1–C2	1.411	1.402	1.399	1.368	1.399	1.393	1.416	1.440
C2–N	1.412	1.438	1.433	1.427	1.468	1.471	1.399	1.391
C5–Cl	1.740	1.736	1.738	1.738	1.738	1.741	1.767	1.731
O–H	0.959	0.958	0.959	0.966	0.966	0.965	0.945	0.991
C4–C1–C2–N	–6.3	–7.6	–5.3	–2.1	–16.5	4.7	–0.9	–9.0
C1–C2–N–C3	–91.0	–86.6	–88.7	–89.2	–76.5	–95.4	–92.3	–88.2
H–O–C4–C1	–31.1	–22.7	–34.3	–42.1	–41.1	–61.1	–31.5	–8.0

a Taken from ref (39).

b Atom numbering taken from Figure 1.

**Table 13:** Selected Geometrical Parameters for the “TWin2” Conical Intersection of SMAC, Using the 6-31G(d,p) Basis Set^b^

	BHHLYP			ωB97X				
parameter	NAC	PC	BP	NAC	PC	BP	CASSCF^a^	XMS-CASPT2
C1–C2	1.409	1.381	1.395	1.368	1.411	1.392	1.416	1.440
C2–N	1.408	1.449	1.346	1.429	1.470	1.469	1.399	1.392
C5–Cl	1.739	1.751	1.738	1.738	1.737	1.738	1.767	1.731
O–H	0.959	1.170	0.957	0.966	0.967	0.966	0.945	0.991
C4–C1–C2–N	7.3	–1.3	2.0	4.0	21.8	–6.1	0.9	9.2
C1–C2–N–C3	88.7	93.2	91.2	88.1	72.3	95.4	92.3	88.1
H–O–C4–C1	31.1	34.8	40.5	40.7	34.6	58.7	31.3	8.1

a Taken from ref (39).

b Atom numbering taken from Figure 1.

**Figure 7:**
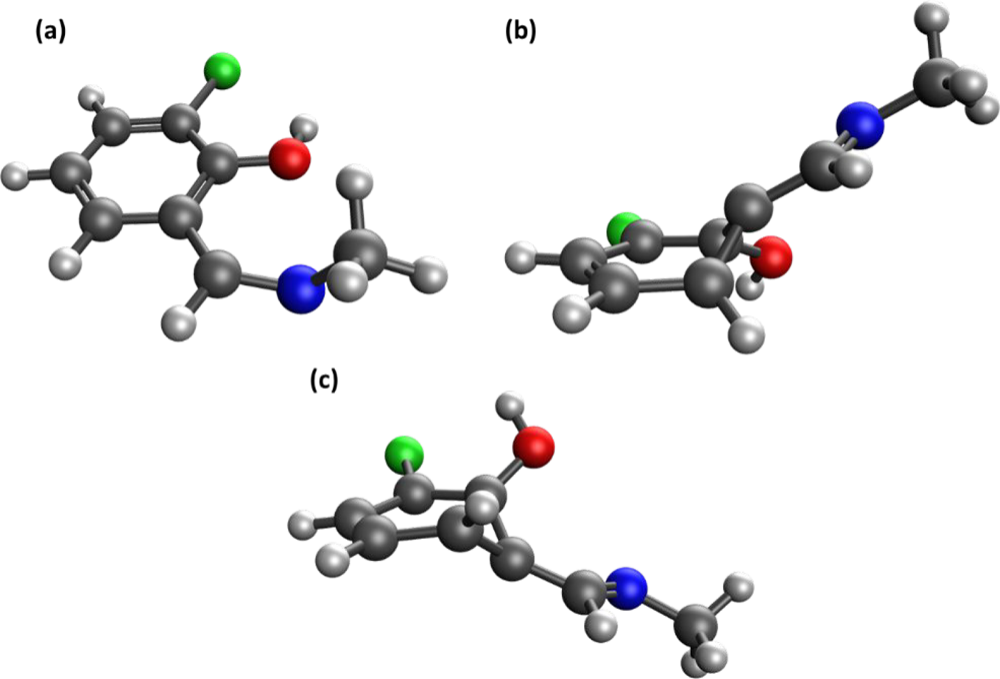
Calculated geometries for the TWout2 conical intersection of SMAC: (a) XMS-CASPT2, (b) PC-BHHLYP, and (c) NAC-BHHLYP.

The XMS-CASPT2 geometries for the “TWout1” and “TWout2” conical intersections are similar to above, but the −OH proton points away from the nitrogen atom (while for “TWin1” and “TWin2” the proton points toward the nitrogen atom; see Figures S8(d) and S8(e)). Each of the SF-TDDFT approaches shows good qualitative agreement with XMS-CASPT2 for “TWout1”, although some of the bond lengths are different by as much as 0.1 Å (Table 14). The ωB97X functional is qualitatively correct for “TWout2” compared to XMS-CASPT2 for each of the methods considered, but PC-BHHLYP and NAC-BHHLYP show very different features (Table 15 and Figure 7), with a significant kink in the ring at position 1 (see Figure 1 for atom numbering).

**Table 14:** Selected Geometrical Parameters for the “TWout1” Conical Intersection of SMAC, Using the 6-31G(d,p) Basis Set^b^

	BHHLYP			ωB97X				
parameter	NAC	PC	BP	NAC	PC	BP	CASSCF^a^	XMS-CASPT2
C1–C2	1.409	1.380	1.397	1.380	1.376	1.380	1.438	1.454
C2–N	1.369	1.468	1.397	1.389	1.473	1.468	1.374	1.361
C5–Cl	1.753	1.751	1.752	1.751	1.752	1.751	1.752	1.745
O–H	0.957	0.965	0.957	0.967	0.965	0.965	0.944	0.969
C4–C1–C2–N	10.2	17.7	7.1	7.7	6.5	17.7	8.9	14.5
C1–C2–N–C3	–98.5	–104.0	–96.9	–99.8	–92.8	–104.0	–97.2	–98.5
H–O–C4–C1	–177.5	–176.5	–177.5	–176.4	–176.6	–176.5	–176.1	–178.2

a Taken from ref (39).

b Atom numbering taken from Figure 1.

**Table 15:** Selected Geometrical Parameters for the “TWout2” Conical Intersection of SMAC, Using the 6-31G(d,p) Basis Set^b^

	BHHLYP			ωB97X				
parameter	NAC	PC	BP	NAC	PC	BP	CASSCF^a^	XMS-CASPT2
C1–C2	1.404	1.404	1.399	1.382	1.384	1.381	1.419	1.454
C2–N	1.282	1.286	1.391	1.388	1.444	1.450	1.379	1.361
C5–Cl	1.719	1.719	1.752	1.751	1.751	1.751	1.778	1.745
O–H	0.956	0.956	0.957	0.966	0.966	0.966	0.943	0.969
C4–C1–C2–N	–31.6	31.6	–4.9	–7.1	–1.7	–1.3	–11.1	–14.5
C1–C2–N–C3	178.3	178.5	92.7	98.8	87.4	87.2	98.8	98.5
H–O–C4–C1	163.8	161.5	173.6	176.9	173.9	173.8	175.8	178.2

a Taken from ref (39).

b Atom numbering taken from Figure 1.

### 4.9 Discussion

The convergence of a conical intersection geometry optimization with SF-TDDFT was often challenging. However, for some of the SF-TDDFT approaches (and basis sets) considered here, the optimization problem seems almost pathological. The two molecules, 4ABN and azomethane, hint at such problems. In both cases, several of the SF-TDDFT methods failed to achieve convergence using the 6-31G(d) basis set (as used in refs (33) and (37)), but the geometry converged smoothly with the larger 6-31G(d,p) basis set. Using this converged geometry as a guess, the 6-31G(d) calculations then swiftly converged, suggesting that the choice of method is relatively insensitive in the immediate region of a conical intersection; the problem is getting to such a region!

Segarra-Marti et al. also demonstrated the need to include dynamical correlation in conical intersection geometry optimizations, by employing XMS-CASPT2.^17^ In some cases, there were significant differences between CASSCF and XMS-CASPT2. We expect the geometrical parameters coincident with the **g** and **h** vectors to be accurately described by CASSCF, but other bond lengths may be expected to be longer. Indeed, we found in a few cases differences between the calculated geometries from XMS-CASPT2 and CASSCF (e.g., 9H-adenine; see Table 5). In addition, the successful application of CASSCF (and, by extension, XMS-CASPT2) is limited by the choice of active space, which itself may be limited by (a) an inexperienced user and/or (b) technical limitations within a given piece of software. The second of these limitations is noted for 9H-adenine, where the authors were restricted to (6,6) for the conical intersection search, despite identifying the “correct” active space as 12 electrons in 10 orbitals.^35^

In some cases in the current work, where the SF-TDDFT-based method failed to give a qualitatively correct geometry for a conical intersection, we tried starting from the CASSCF and/or XMS-CASPT2 geometries and reoptimizing, e.g., 4ABN (Figure 2) and 2,4,6-octatriene (Figure 5). In each of these cases, the combination of algorithm and functional (plus basis set) led the geometry away from that determined by XMS-CASPT2 to the qualitatively incorrect structures observed. This was mainly observed when nonadiabatic coupling terms were neglected (i.e., the PC or BP approaches) or in the case of the ESIPT MECP of SMAC, with NAC-ωB97X. This agrees well with the work of Herbert et al.,^48^ who recommend the use of BHHLYP when identifying MECP structures with SF-TDDFT.

Given in Table 16 are the mean deviations, mean unsigned deviations, and maximum errors for the geometrical parameters. While the mean deviations look very encouraging, the mean unsigned error is a more realistic measure for each of the geometrical parameters. The maximum errors are due to the few cases noted above, in particular the failure of PC-ωB97X to correctly describe the 2,4,6-octatriene MECP. While spin-contamination can be an issue with SF-TDDFT, in the case of 2,4,6-octatriene with ⟨*S*^2^⟩ values stay well below the default thresholds (1.20), and the failure can thus be attributed to the penalty-constrained projection algorithm, rather than SF-TDDFT. While NAC-ωB97X fails to correctly describe the ESIPT MECP of SMAC, NAC-BHHLYP matches the XMS-CASPT2 geometry. Once again, spin-contamination is not an issue here; Herbert et al. recommend the use of BHHLYP when using SF-TDDFT, and our results confirm this. We also calculated *s^x^* and *s^y^* tilt parameters,^48^ which confirmed each of the MECP geometries obtained in this study had a peaked topology.

**Table 16:** Mean Deviation, Mean Unsigned Deviations, and Maximum Deviations for CASSCF and Each of the SF-TDDFT Methods Considered in This Work

			BHHLYP			ωB97X		
		CASSCF	NAC	PC	BP	NAC	PC	BP
bonds	mean dev	–0.006	–0.008	0.001	0.001	–0.004	0.005	–0.001
	MUE	0.019	0.024	0.032	0.034	0.024	0.032	0.026
	Max	0.111	0.119	0.185	0.226	0.105	0.113	0.107
angles	mean dev	0.9	–1.8	–1.0	0.6	–1.5	–3.8	–0.4
	MUE	2.0	3.2	2.9	4.5	3.4	6.4	4.3
	Max	5.6	16.1	16.1	18.8	16.1	16.1	16.1
dihedrals	mean dev	1.3	1.2	1.7	–0.8	1.5	1.6	–0.7
	MUE	7.3	13.9	13.9	13.2	13.9	18.4	18.3
	Max	34.9	93.8	93.8	93.8	98.8	102.1	98.7

The BP-BHHLYP and PC-BHHLYP approaches show generally good agreement with the XMS-CASPT2 computed MECP geometries; they also closely match the NAC-BHHLYP geometries, with less computational effort required. The BP-ωB97X approach also shows reasonable agreement, but the PC-ωB97X shows some significant deviations. The relative energetics for each molecule considered are given in Tables S2–S20 and Figure S9 in the Supporting Information. The qualitative picture here is generally good in comparison to the XMS-CASPT2 energies, with a few exceptions (related to the qualitative disagreement of the MECP geometries). For 9H-adenine, only CASSCF qualitatively matches the gap relative to the vertical excitation energy for the MECP energy; this is lower than the vertical excitation energy, while all of the SF-TDDFT approaches give a relative energy higher than the vertical excitation energy. The deviation from the XMS-CASPT2 energies in most cases is within 1 eV with largely the correct qualitative trend but with relatively low quantitative accuracy. We note that NAC-BHHLYP most closely follows the XMS-CASPT2 relative energy values (Figure S9), while PC-BHHLYP and PC-ωB97X both outperform the branching plane update method.

## 5 Conclusions

We have studied different SF-TDDFT-based approaches for optimizing conical intersection geometries and compared them to the XMS-CASPT2 method. NAC-BHHLYP is the most reliable method for calculating the MECP geometries, but BP-BHHLYP and PC-BHHLYP also demonstrate good agreement, while having a substantially reduced computational cost in comparison to NAC-BHHLYP. Keal et al.^49^ concluded that the penalty-constrained approach should only be used where full nonadiabatic coupling terms cannot be calculated; while we have demonstrated that PC-BHHLYP appears to be reliable in most situations, we would also agree that NAC-BHHLYP should be employed where possible. For reasonable relative energetics, only the NAC-BHHLYP approach can be recommended; thus, initial MECP optimization could be performed by PC-BHHLYP and refined by NAC-BHHLYP.

## Supporting Information Available

XMS-CASPT2 3D-rendered geometries and calculated energies (PDF)
