# Supplementary material for: Comparison of Spin-Flip TDDFT-Based Conical Intersection Approaches with XMS-CASPT2
Source: J Chem Theory Comput. 2020 Apr 17;16(5):3253–63. doi: 10.1021/acs.jctc.9b00917 (PMC8279405; doi:10.1021/acs.jctc.9b00917)
Supplement: Supplementary file 1 — ct9b00917_si_001.pdf [file ct9b00917_si_001.pdf]

A Comparison of Spin-Flip TDDFT-Based Conical Intersection Approaches with CASSCF

*Max Winslow, Warren B. Cross and David Robinson**

Department of Chemistry and Forensics, School of Science and Technology, Nottingham Trent University, Clifton Lane, Nottingham, NG11 8NS, United Kingdom

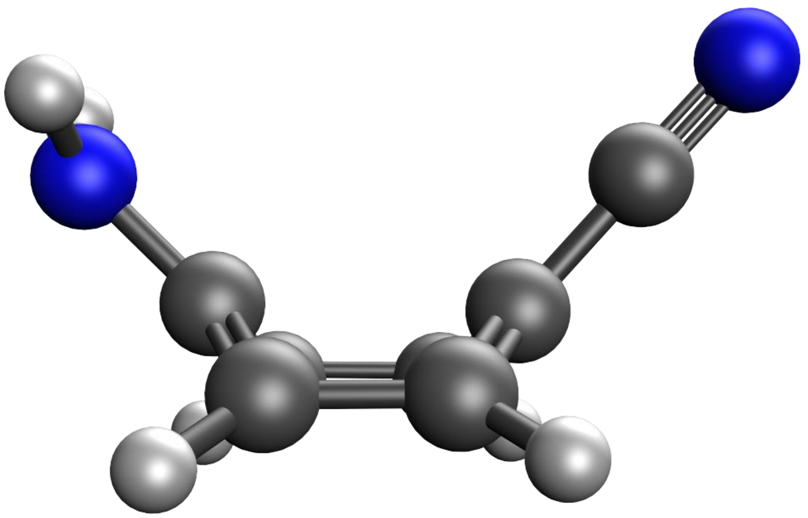

Figure S1. 4ABN conical intersection calculated with XMS-CASPT2/6-31+G(d)

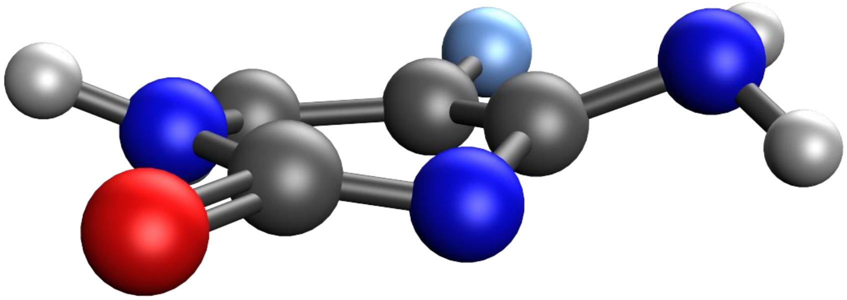

Figure S2. 5FC conical intersection calculated with XMS-CASPT2/6-31G(d)

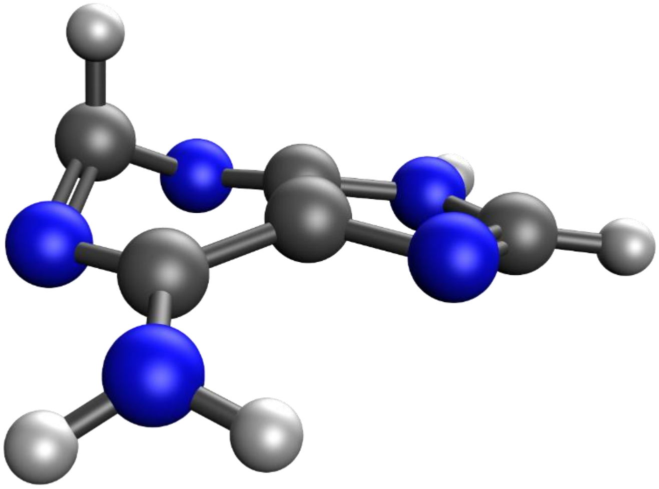

Figure S3(a). 9H-adenine nπ* conical intersection calculated with XMS-CASPT2/6-31G(d,p)

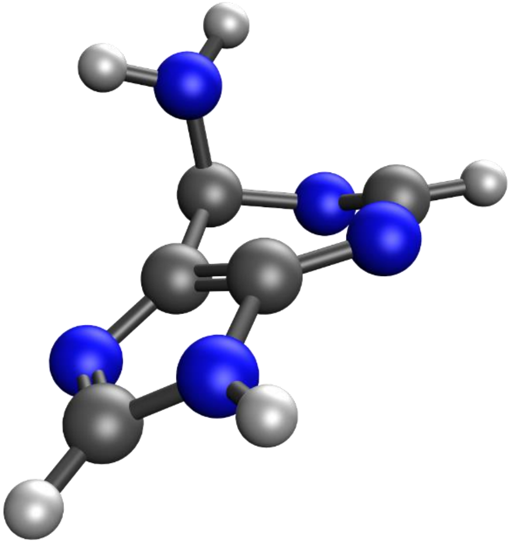

Figure S3(b). 9H-adenine ππ* (^1L_b) conical intersection calculated with XMS-CASPT2/6-31G(d,p).

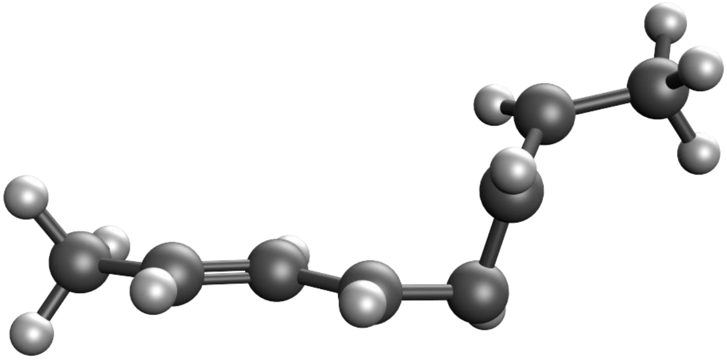

Figure S4. 2,4,6-octatriene conical intersection calculated with XMS-CASPT2/6-31+G(d).

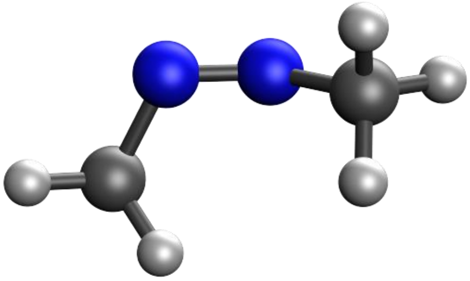

Figure S5. Azomethane conical intersection calculated using XMS-CASPT2/6-31G(d).

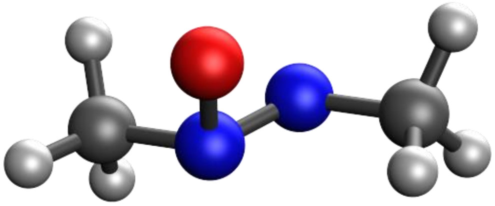

Figure S6. Azoxymethane conical intersection calculated using XMS-CASPT2/6-31G(d).

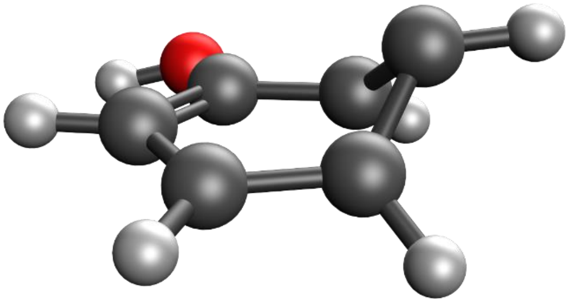

Figure S7. Phenol conical intersection calculated using XMS-CASPT2/6-31G(d,p).

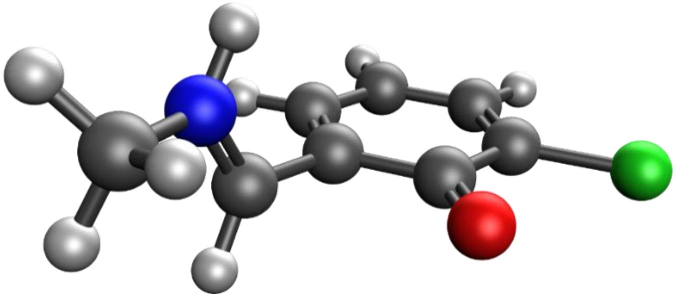

Figure S8(a). SMAC ESIPT conical intersection calculated using XMS-CASPT2/6-31G(d,p).

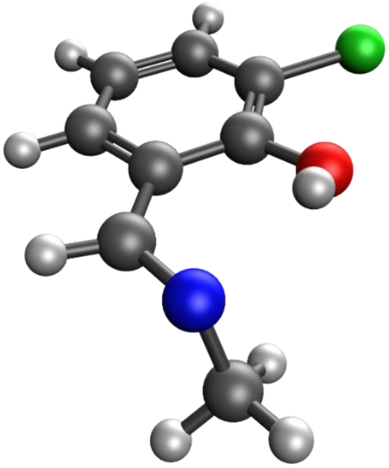

Figure S8(b). SMAC TWin1 conical intersection calculated using XMS-CASPT2/6-31G(d,p).

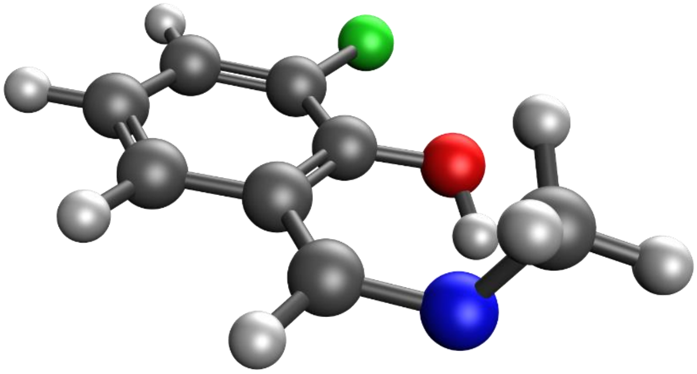

Figure S8(c). SMAC TWin2 conical intersection calculated using XMS-CASPT2/6-31G(d,p).

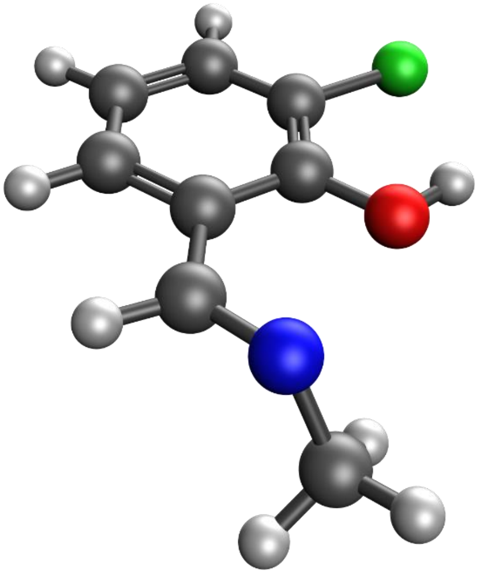

Figure S8(d). SMAC TWout1 conical intersection calculated using XMS-CASPT2/6-31G(d,p).

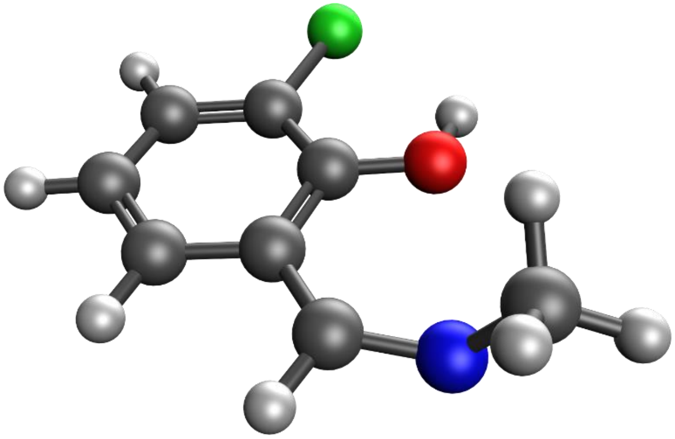

Figure S8(e). SMAC TWout2 conical intersection calculated using XMS-CASPT2/6-31G(d,p).

Molecule	CASSCF	BHHLYP Branched	BHHLYP Penalty	BHHLYP NAC	ωB97X Branched	ωB97X Penalty	ωB97X NAC
Fulvene	✓ (green)	✓ (green)	✓ (green)	✓ (green)	✓ (green)	✓ (green)	✓ (green)
4ABN	✓ (green)	✓ (amber)	✓ (amber)	✓ (amber)	✓ (amber)	✓ (amber)	✓ (amber)
5FC	✓ (green)	✓ (amber)	✓ (amber)	✓ (amber)	✓ (amber)	✗ (red)	✓ (amber)
9H-adenine nπ*	✓ (green)	✓ (green)	✓ (green)	✓ (green)	✓ (green)	✓ (amber)	✓ (green)
9H-adenine ππ*	✓ (amber)	✓ (green)	✓ (green)	✓ (green)	✓ (green)	✓ (green)	✓ (green)
2,4,6-octatriene	✓ (green)	✓ (green)	✓ (green)	✓ (green)	✓ (green)	✗ (red)	✓ (green)
Azomethane	✓ (green)	✓ (green)	✓ (green)	✓ (green)	✓ (green)	✓ (green)	✓ (green)
Azoxymethane	✓ (green)	✓ (amber)	✓ (amber)	✓ (green)	✓ (amber)	✓ (amber)	✓ (amber)
Phenol	✓ (green)	✓ (amber)	✓ (green)	✓ (green)	✓ (green)	✓ (green)	✓ (green)
SMAC ESIPT	✓ (green)	✗ (red)	✓ (amber)	✓ (amber)	✗ (red)	✓ (amber)	✗ (red)
SMAC TWin1	✓ (green)	✓ (green)	✓ (green)	✓ (green)	✓ (green)	✓ (green)	✓ (green)
SMAC TWin2	✓ (green)	✓ (green)	✓ (green)	✓ (green)	✓ (green)	✓ (amber)	✓ (green)
SMAC TWout1	✓ (green)	✓ (green)	✓ (green)	✓ (green)	✓ (green)	✓ (green)	✓ (green)
SMAC TWout2	✓ (green)	✓ (green)	✗ (red)	✗ (red)	✓ (green)	✓ (green)	✓ (green)

Table S1. Schematic overview of the agreement between the different TDDFT methods and XMS-CASPT2. Key: Green tick – good qualitative and quantitative agreement; amber tick – good qualitative agreement; red cross – poor qualitative agreement.

	XMS-CASPT2	CASSCF	BHHLYP NAC	BHHLYP PC	BHHLYP BP	ωB97X NAC	ωB97X PC	ωB97X BP
VE	3.39	4.07		3.70			2.74	
0-0	2.44	2.60		3.13			3.24	
S_1/S_0	2.41	2.46	2.95	2.94	2.95	3.14	3.13	3.18

Table S2. Relative energetics of fulvene. All energies are relative to the S_0 minimum.

	XMS-CASPT2	CASSCF	BHHLYP NAC	BHHLYP PC	BHHLYP BP	ωB97X NAC	ωB97X PC	ωB97X BP
VE	4.06	4.68		5.32			5.08	
0-0	3.95	4.09		5.02			4.94	
S_1/S_0	4.07	5.15	4.87	5.04	4.88	4.84	4.83	4.94

Table S3. Relative energetics of 4ABN. All energies are relative to the S_0 minimum.

	XMS-CASPT2	CASSCF	BHHLYP NAC	BHHLYP PC	BHHLYP BP	ωB97X NAC	ωB97X PC	ωB97X BP
VE	4.37	5.31		5.36			5.30	
0-0	3.75	3.93		4.78			4.85	
S_1/S_0	4.55	4.55	4.95	4.81	4.95	6.00	5.71	5.99

Table S4. Relative energetics of 5FC. All energies are relative to the S_0 minimum.

	XMS-CASPT2	CASSCF	BHHLYP NAC	BHHLYP PC	BHHLYP BP	ωB97X NAC	ωB97X PC	ωB97X BP
VE	5.13	5.43		4.80			4.92	
0-0	4.39	4.63		5.06			4.89	
S_1/S_0	4.56	4.46	5.33	5.24	5.26	5.27	6.90	5.30

Table S5. Relative energetics of 9H-adenine ($^1\pi\pi^*$). All energies are relative to the S_0 minimum.

	XMS-CASPT2	CASSCF	BHHLYP NAC	BHHLYP PC	BHHLYP BP	ωB97X NAC	ωB97X PC	ωB97X BP
VE	5.73	6.36		5.73			5.73	
0-0	4.51	5.12		5.07			4.89	
S_1/S_0	4.12	4.59	5.75	7.33	7.33	6.26	7.14	6.85

Table S6. Relative energetics of 9H-adenine ($^1n\pi^*$). All energies are relative to the S_0 minimum.

	XMS-CASPT2	CASSCF	BHHLYP NAC	BHHLYP PC	BHHLYP BP	ωB97X NAC	ωB97X PC	ωB97X BP
VE	3.23	3.45		3.30			3.43	
0-0	2.87	3.06		2.89			2.91	
S_1/S_0	2.76	2.82	2.97	2.97	2.97	3.05	3.23	3.24

Table S7. Relative energetics of azomethane. All energies are relative to the S_0 minimum.

	XMS-CASPT2	CASSCF	BHHLYP NAC	BHHLYP PC	BHHLYP BP	ωB97X NAC	ωB97X PC	ωB97X BP
VE	4.66	4.45		4.09			4.21	
0-0	3.88	2.95		3.30			2.93	
S_1/S_0	3.87	2.91	3.37	3.84	3.86	4.15	4.63	4.38

Table S8. Relative energetics of azoxymethane. All energies are relative to the S_0 minimum.

	XMS-CASPT2	CASSCF	BHHLYP NAC	BHHLYP PC	BHHLYP BP	ωB97X NAC	ωB97X PC	ωB97X BP
VE	5.78	5.58		4.83			5.04	
0-0	5.51	5.21		4.83			4.89	
S_1/S_0	3.99	3.72	2.46	2.44	7.85	4.28	4.48	7.43

Table S9. Relative energetics of 2,4,6-octatriene. All energies are relative to the S_0 minimum.

	XMS-CASPT2	CASSCF	BHHLYP			ωB97X		
			NAC	PC	BP	NAC	PC	BP
VE	4.82	4.93		5.50			5.26	
0-0	4.64	4.73		5.55			5.40	
S_1/S_0	4.95	5.45	5.46	5.65	5.46	5.63	5.95	5.63

Table S10. Relative energetics of phenol. All energies are relative to the S_0 minimum.

	XMS-CASPT2	CASSCF	BHHLYP			ωB97X		
			NAC	PC	BP	NAC	PC	BP
$S_0(\alpha)$	0.37	0.27		0.25			0.27	
$S_0(\beta)$	0.00	0.44		0.00			0.00	
$S_0(\gamma)$	0.50	0.60		0.43			0.45	
$S_0(\delta)$	0.37	0.33		0.44			0.42	
$S_0(\zeta)$	0.15	0.00		0.04			0.17	
$S_1(\alpha)$	4.58	4.75		3.38			3.27	
$S_1(\beta)$	2.72	3.61		3.61			3.68	
$S_1(\gamma)$	2.98	3.71		3.89			2.84	
$S_1(\delta)$	4.63	4.96		3.35			3.18	
$S_1(\zeta)$	4.34	4.46		3.30			3.19	
ESIPT	2.35	3.08	3.10	3.08	4.22	5.03	3.78	5.05
TWin1	2.15	3.62	3.25	3.24	3.52	3.75	4.59	4.77
TWin2	2.15	3.62	3.25	3.24	3.54	3.75	4.59	4.76
TWout1	2.38	3.66	3.16	3.15	3.48	3.59	4.59	4.71
TWout2	2.38	3.66	5.62	5.61	3.48	3.59	4.60	4.62

Table S11. Relative energetics of SMAC. All energies are relative to the S_0 minimum.

	XMS-CASPT2	CASSCF	BHHLYP			ωB97X		
			NAC	PC	BP	NAC	PC	BP
VE								
0-0	-0.95	-1.47		-0.57			-0.37	
S_1/S_0	-0.98	-1.61	-0.74	-0.76	-0.74	-0.47	-0.48	-0.43

Table S12. Relative energetics of fulvene. All energies are relative to the vertical excitation energy.

	XMS-CASPT2	CASSCF	BHHLYP			ωB97X		
			NAC	PC	BP	NAC	PC	BP
VE								
0-0	-0.11	-0.59		-0.30			-0.14	
S_1/S_0	0.01	0.47	-0.45	-0.28	-0.44	-0.24	-0.25	-0.14

Table S13. Relative energetics of 4ABN. All energies are relative to the vertical excitation energy.

	XMS-CASPT2	CASSCF	BHHLYP			ωB97X		
			NAC	PC	BP	NAC	PC	BP
VE								
0-0	-0.62	-1.38		-0.58			-0.45	
S_1/S_0	0.17	-0.76	-0.41	-0.55	-0.41	0.70	0.41	0.69

Table S14. Relative energetics of 5FC. All energies are relative to the vertical excitation energy.

	XMS-CASPT2	CASSCF	BHHLYP			ωB97X		
			NAC	PC	BP	NAC	PC	BP
VE								
0-0	-0.74	-0.81		0.26			-0.03	
S_1/S_0	-0.57	-0.97	0.53	0.44	0.46	0.35	0.34	0.38

Table S15. Relative energetics of 9H-adenine ($^1\pi\pi^*$). All energies are relative to the vertical excitation energy.

	XMS-CASPT2	CASSCF	BHHLYP			ωB97X		
			NAC	PC	BP	NAC	PC	BP
VE								
0-0	-1.21	-1.24		-0.66			-0.84	
S_1/S_0	-1.61	-1.76	0.02	1.60	1.60	0.53	1.41	1.12

Table S16. Relative energetics of 9H-adenine ($^1n\pi^*$). All energies are relative to the vertical excitation energy.

	XMS-CASPT2	CASSCF	BHHLYP			ωB97X		
			NAC	PC	BP	NAC	PC	BP
VE								
0-0	-0.36	-0.39		-0.41			-0.52	
S_1/S_0	-0.47	-0.63	-0.33	-0.33	-0.33	-0.38	-0.20	-0.19

Table S17. Relative energetics of azomethane. All energies are relative to the vertical excitation energy.

	XMS-CASPT2	CASSCF	BHHLYP			ωB97X		
			NAC	PC	BP	NAC	PC	BP
VE								
0-0	-0.78	-1.50		-0.79			-1.28	
S_1/S_0	-0.79	-1.54	-0.72	-0.25	-0.23	-0.06	0.42	0.17

Table S18. Relative energetics of azoxymethane. All energies are relative to the vertical excitation energy.

	XMS-CASPT2	CASSCF	BHHLYP			ωB97X		
			NAC	PC	BP	NAC	PC	BP
VE								
0-0	-0.27	-0.36		0.00			-0.15	
S_1/S_0	-1.79	-1.85	-0.28	-0.29	-0.27	-0.76	-0.57	-0.04

Table S19. Relative energetics of 2,4,6-octatriene. All energies are relative to the vertical excitation energy.

	XMS-CASPT2	CASSCF	BHHLYP			ωB97X		
			NAC	PC	BP	NAC	PC	BP
VE								
0-0	-0.18	-0.20		0.05			0.14	
S_1/S_0	0.13	0.52	-0.04	0.15	-0.04	0.37	0.69	0.37

Table S20. Relative energetics of phenol. All energies are relative to the vertical excitation energy.

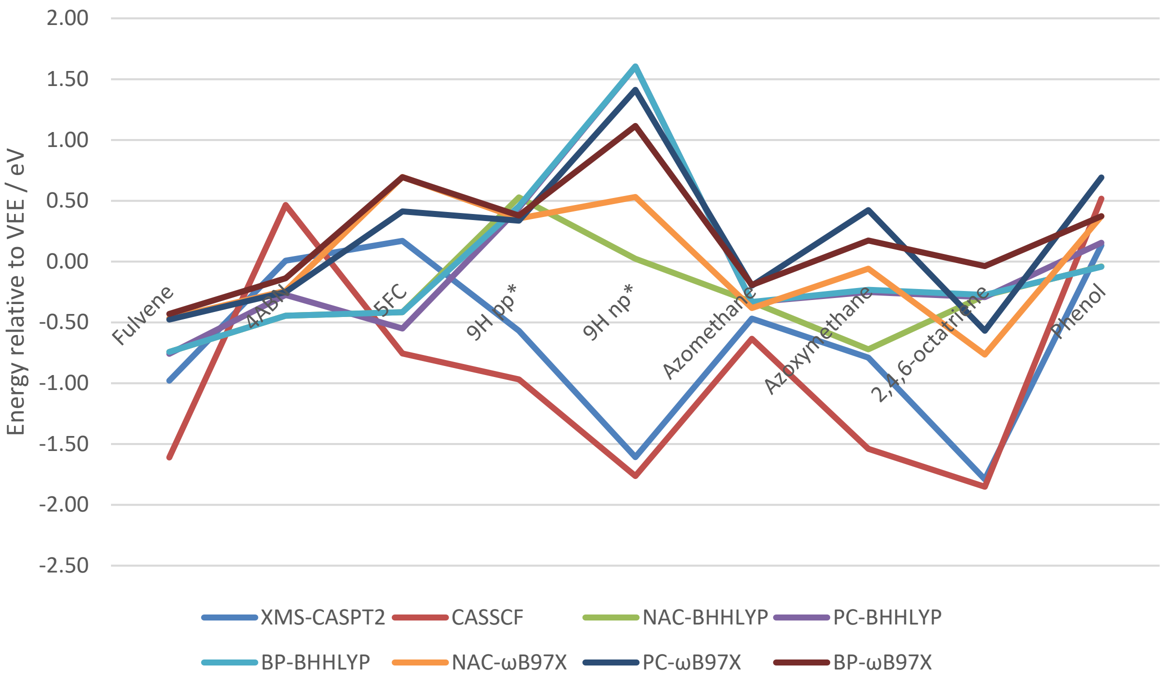

Figure S9. Relative energies of the calculated MECPs relative to the vertical excitation energy (VEE) for all molecules except SMAC.
